# Influence of Tunneled Hemodialysis-Catheters on Inflammation and Mortality in Dialyzed Patients

**DOI:** 10.3390/ijerph18147605

**Published:** 2021-07-16

**Authors:** Rodolfo Crespo-Montero, Victoria E. Gómez-López, Fátima Guerrero-Pavón, Andrés Carmona-Muñoz, Manuel Romero-Saldaña, Antonio Ranchal-Sanchez, Pedro Aljama-García

**Affiliations:** 1 Department of Nursing, Faculty of Medicine and Nursing, University of Córdoba, 5000 Córdoba, Spain; z92rosam@uco.es (M.R.-S.); en1rasaa@uco.es (A.R.-S.); 2 Nephrology Service, Reina Sofía University Hospital, 5000 Córdoba, Spain; rodo.crespo@gmail.com; 3 Maimonides Institute of Biomedical Research of Cordoba, 5000 Córdoba, Spain; fatima.guerrero@imibic.org (F.G.-P.); carmonacarpio29@hotmail.com (A.C.-M.); paljamag@gmail.com (P.A.-G.)

**Keywords:** central venous catheter, fistula, hemodialysis, inflammatory status, mortality

## Abstract

Older age and comorbidities in hemodialysis patients determines the use of tunneled catheters as vascular access despite their reported clinical and mortality disadvantages. This prospective matched study analyzes the impact of permanent catheters on inflammation and mortality in hemodialysis patients; We studied 108 patients, 54 with AV-fistula (AVF) and 54 with indwelling hemodialysis catheters (HDC) matched by sex, age, diabetes and time under renal-replacement therapy comparing dialysis efficacy, inflammation and micro-inflammation parameters as well as mortality. Cox-regression analysis was applied to determine predictors of mortality, HDC patients presented higher C-reactive-protein (CRP) blood levels and percentage of pro-inflammatory lymphocytes CD14+/CD16+ with worse dialysis-efficacy parameters. Thirty-six-months mortality appeared higher in the HDC group although statistical significance was not reached. Age with a Hazard Ratio (HR) = 1.06, hypoalbuminemia (HR = 0.43), hypophosphatemia (HR = 0.75) and the increase in CD14+/CD16+ monocyte count (HR = 1.02) were predictors of mortality; elder patients dialyzing through HDC show increased inflammation parameters as compared with nAVF bearing patients, although they do not present a significant increase in mortality when matched by covariates. Increasing age and percentage of pro-inflammatory monocytes as well as decreased phosphate and serum-albumin were predictors of mortality and indicate the main conclusions or interpretations.

## 1. Introduction

The vascular access (VA) for hemodialysis (HD) constitutes a critical issue in end-stage renal failure (ESRF) patients. It is commonly accepted that native arterio-venous fistulae (nAVF) are the elective VAs accordingly to their reduced infection ratios and better long-term permeability [1,2] but, nevertheless, the use of indwelling venous central catheters (VCCs) has increased progressively [3,4], especially in elderly comorbid patients [5].

The “fistula first” initiative reached success changing this trend^6^ and in recent years large studies like the DOPSS (Dialysis Outcomes and Practice Patterns Study) have demonstrated a reduction in hemodialysis catheter (HDC) use in a majority of participating countries [6]. However, nAVFs are not always feasible: increasing age, greater cardiovascular comorbidity and the presence of diabetes mellitus in the dialysis patient population yields significant percentages of patients nowadays dialyzing through indwelling VCCs [7].

Besides the “fistula first” initiative, several vascular access guidelines recommend minimizing HDC use for chronic dialysis [8]. These best practice recommendations rely in the evidence of VCC use in hemodialysis as an independent mortality predictor [9]. In fact, there is ample published evidence over the close relationship between HDC use and dialysis-related mortality [10,11]. The increase in mortality-ratio as compared with nAVFs has been put in relation with an increase in invasive infections, low-quality renal replacement delivery or a subclinical inflammatory state, associated with the VCC [12,13] suggesting a determinant role in the patient’s inflammatory response as compared with nAVFs [14]. It must be stated though that most studies analyzing morbimortality in VCC-bound patients have an observational design, limiting their ability to address eventual cause-and-effect relationships which abrogates the chance to eradicate nAVF-indication bias. Moreover, the current evidence favoring nAVFs superiority over HDCs precludes the implementation of randomized controlled trials (RCTs) comparing both VAs for ethical reasons. In this context, we decided to start this paired study matching for variables settled in the scientific literature for their impact on the inflammatory state and influence in survival of hemodialysis patients with similar baseline profiles dividing them by the use of HDC vs. nAVF.

Therefore, the objectives of the study are: (1) To compare the inflammation status amongst hemodialysis patients with HDC vs. nAVF. (2) To compare survival between both groups analyzing mortality-related factors.

## 2. Materials and Methods

### 2.1. The Scope of the Study

The study was carried out within the area of influence of the Nephrology Department at Reina Sofía University Hospital of Córdoba (Spain). It was started started the 1st of July, 2016 with the baseline analysis of the sample concluding the 31st of December, 2019 with a 36 month follow up for the survival analysis.

### 2.2. Population, Sampling and Inclusion Criteria

A total of 398 patients were under hemodialysis therapy, from which 324 (81.4%) carried a nAVF, 72 (18.1%) a tunneled HDC and 2 (0.5%) a prosthetic AVF.

Inclusion criteria for tunneled VCC were:

At least 3 months bearing an indwelling tunneled VCC.

No past history of catheter-related infection in the previous 3 months.

Not having been diagnosed with any systemic condition potentially associated with an acute or chronic inflammatory state.

These criteria excluded nine patients, leaving a total of 65 eligible patients.

For nAVF patients eligible for matching, inclusion criteria were:

nAVF at least 3 months in use.

No past history of nAVF-related infection within the last 3 months.

Not having been diagnosed with any systemic condition potentially associated with an acute or chronic inflammatory state.

#### 2.2.1. Dialysis Scheme

Patients received hemodialysis as prescribed per clinical and analytical requirements, on a three-weekly basis, 3 to 5 h per session, using 5008 S or 5008 Cordiax devices (Fresenius Fresenius Medical Care, Bad Homburg, Germany) equipped with OCM biosensors (On-line clearance monitoring), non-invasively measuring ionic dialisance equivalent to the clearance of urea (K). The systematic register of K multiplied by the elapsed time allows the procurement of Kt, which constitutes a mean to effectively measure the provided dose of dialysis, expressed in liters, and the Kt/V (clearance of urea*time in dialysis/Urea distribution volume).

All the subjects had sinthetic membrane dialyzers (Helixona^®^, Fresenius Medical Care, Bad Homburg, Germany). Indwelling HDCs were made of polyuretane Equistream^®^ (Bard Access Systems, Salt Lake City, UT, USA). Forty-nine (91%) patients had the CVCt placed in the right internal jugular vein and 5 (9%) in the left internal jugular vein.

#### 2.2.2. Nursing Care of HDCs

Nursing care and management of HDCs was similar for all the patients. Connection and disconnection procedures to the extracorporeal circuit lines followed the recommendations from the Vascular Access Guidelines of the Spanish Society of Nephrology [8]. These procedures emphasized the prevention of infections (sterile single-use material use, surgical-mask wearing for patient and nurse during connection and disconnection, and in every needed HDC handling during the dialysis session, and 1% sodium-heparine sealing as well as 2% aqueous clorhexidine antiseptic cleansing for the skin exit orifice).

### 2.3. Study Design

Prospective observational matched cohort study of patients with indwelling HDCs or nAVFs, in concordance with the most influential covariables regarding the inflammatory status and mortality in these patients: age, sex, time in hemodialysis (from the moment of being established in the therapy up to the beginning of the study) and Diabetes Mellitus (DM). Matching for age was elected for ±10 years and for time-in-dialysis ±60 months. The matching was computerized with the Nephrology Departmnet database, curating data, verifying one-by-one matches and checking equality of the groups for adjusting covariables.

Baseline inflammatory and clinical parameters for the patients were determined. Later, follow up was carried for survival analysis. Follow-up concluded with the death of the subject or the termination of the study after 36 months.

### 2.4. Study Variables and Labs

Inflammation, microinflammation and mortality markers were taken as variables of outcome.

Inflammation was determined by the following parameters: C-reactive-protein (CRP), Albumin and Ferritin. Microinflammation was measured with the percentage of pro-inflammatory circulant monocyte subpopulation in peripheral blood CD14+/CD16− and CD14+/CD16+.

#### Cell Cultures

Immunolabelling and flow cytometry were performed in whole blood to avoid centrifugation and washing steps which can lead to artifactual platelet activation. Aliquots of whole blood (100 μL) were incubated with peridinin chlorophyll protein-conjugated monoclonal anti-CD14 (M5E2), FITC-conjugated anti-CD16 (3G8) and isotype matched controls for 20 min (min) at room temperature (RT). Thereafter, samples were fixed and the red cells lysed by the addition of 500 μL of FACS-Lyse solution (Becton Dickinson). Both antibodies and the appropriate isotype controls were purchased from BD Biosciences (San Jose, CA). Flow cytometry was performed on a FACSCalibur (BD Biosciences) using CellQuest. Monocytes were identified based on their forward and side scatter characteristics. For each measurement, a minimum of 2500 monocytes were collected. The percentage of CD14+/CD16− and CD14+/CD16+ monocytes was calculated by subtracting nonspecifically stained cells, as identified in the isotype control histogram.

Last hemodialysis session data (Blood Pressure, Kt/V, Kt, length of dialysis procedure, blood flow, volume of dialyzed blood per session, dialysis modality (On-Line Hemodialfiltration vs. conventional hemodialysis)) were collected simultaneously with the blood sampling to determine the degree of the inflammation as well as other parameters such as Hemoglobin and Serum Phospate and Potassium and Parathyroid Hormone. Comorbidity was also estimated by the Charlson Comorbidity Index (CCI). NAVF-related infections were documented as well as any-cause hospitalization periods. Total time with an indwelling HDC was also registered.

Furthermore, in the patients with CVCt, the following were collected: the time they had been with the catheter, episodes of dysfunction (Blood Flow < 300 mL/min), intraluminal fibrinolytic treatments and catheter replacements; and in patients with AVF, the following was collected: blood recirculation > 15%, (BTM Blood Temperature Monitor) and a sustained decrease in the Kt index (OCM biosensor) for the detection of stenosis [8].

### 2.5. Legal and Ethical Issues

All patients were informed about the purpose of the study and signed an informed consent sheet before entering into the study. Favorable Córdoba Ethics Committee approval was obtained in advance (3 December 2015, Ref. 2999).

### 2.6. Statistical Analysis

Qualitative variables were represented as frequency charts and their percentages whilst quantitative variables were expressed as mean ± standard deviation or median and interquartilic range, depending on their normal-non normal distribution after contrasting with the Kolmogorov–Smirnov test. Means were compared by Student’s *t* test whilst non-parametric Mann–Whitney’s test was employed when applicable. Survival was estimated by the Kaplan–Meyer test and compared between groups using the Log Rank test. Proportional Risks Cox Regression Multivariate Analysis was carried for the time to the outcome variable as well as raw Hazard Ratio (HR) for each explanatory variable. An adjusted model was created from the statistically significant variables. The probability of an α error of below 5% *(p* < 0.05) was considered statistically significant for all the statistical analyses. The confidence interval was calculated at 95%. For the statistical analysis, IBM SPSS Statistics 22.0 software (IBM, Chicago, IL, USA) was used.

## 3. Results

### 3.1. Study Sample Description

Fifty-four patients with indwelling HDC were matched with another 54 patients bearing nAVFs. A final sample including 108 patients was obtained. Table 1 represents data with matching covariables and baseline etiology from both groups of patients.

HDCs were carried by the patients a median of 32 months (Q1 = 18 months and Q3 = 60 months). No relationship was found between this time and the rest of the variables.

There were no catheter-related bacteriemias during the study although it was suspected in two hospital admissions due to fever of unknown origin. There were 30 (median = 4, range between 2 and 7) episodes of dysfunction due to blood flow deficit (<300 mL/min) that required 20 intraluminal fibrinolytic treatments with urokinase^®^ in patients with CVCt.

In patients with AVF, there were no episodes of blood recirculation or a sustained decrease in Kt, so there was no evidence of any alteration that would suggest stenosis of the fistulas.

### 3.2. Inflammation and Microinflammation Parameters

When comparing seldom inflammatory parameters amongst groups, patients carrying HDCs showed a statistically significant increase in CRP and serum Ferritin, as well as a decreased serum albumin, as compared with nAVF bearing patients. Respecting microinflammation, the percentage of proinflammatory monocytes CD14+/CD16+, was significantly increased in patients with HDCs as compared to patients with nAVFs as is shown in Table 2.

In Supplementary Figure S1, we represent flow cytometry charts after determining monocytes and CD14+/CD16+ expression with the dot-diagram in HDC and nAVF bearing patients.

### 3.3. Analytic Values, Dialysis Efficacy, Comorbidity and Hospital Admissions

Patients with HDcs had a lower hemoglobin (Hb) and higher serum phosphate and potassium, the latest showing statistical significance. Patients with CVCt had lower levels of systolic blood pressure and hemoglobin and higher serum phosphorus and potassium, although with significant differences only for potassium. PTH levels were higher in patients with CVCt, although without significant differences.

nAVF bearing patients presented better dialysis efficacy parameters, reaching statistical significance in the treated blood volume per session, dialysis arterial blood flow, Kt/V and Kt, although 91% of the patients from both groups had a Kt/V > 1.3; (99% of nAVF and 92% of HDC). When the Kt/V was compared amongst diabetic and non-diabetic patients, there were no significant differences (1.92 ± 0.46 vs. 1.81 ± 0.48, *p* = 0.261) but reaching significance the differences between men and women (2.0 ± 0.48 vs. 1.73 ± 0.41, respectively, *p* = 0.003). Mean Kt in women was 52.33 ± 8.59 L with a 95% CI (50.03–54.63). In men, it was 53.39 ± 9.16 L with 95% CI (50.72–56.05).

There were no differences in comorbidity between groups and despite a higher total number of hospital-days in HDC carrying patients, it did not reach statistical significance as may be seen in Table 3.

### 3.4. Survival Analysis

There were 48 deaths (44.4%) during follow up (25 women and 23 men). Global and vascular access-specific group causes of death are described in Table 4. No patient required a kidney transplant. No study patients were censored for transplantation during the 36-month follow-up period.

**Figure 1:**
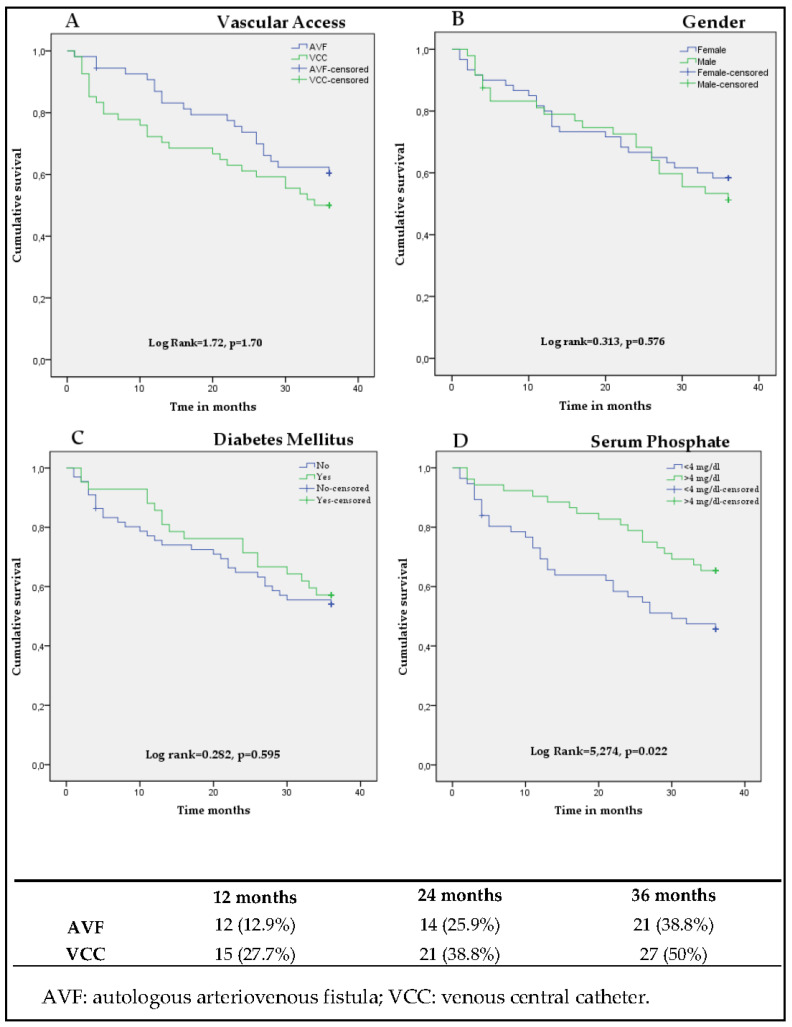
(**A**–**D**). Kaplan Meyer curves, comparing survival according to venous access, gender, diabetes mellitus and serum phosphorus levels (<4 mg/dL or >4 mg/dL) using the Log Rank test. The percentages of deaths at 12, 24, 36 months are described for patients with AVF and VCC.

With the Log Rank test, differences were non-significant between nAVF and HDC groups despite there being an 11.1% higher 36-month mortality in the HDC group (Figure 1A).

Moreover, there were neither significant differences in survival between men and women (Figure 1B) nor amongst diabetics and non-diabetics (Figure 1C).

Serum phosphate (P) was split into two groups by the median (4 mg/dL): patients with *p* < 4 mg/dL, and patients with *p* > 4 mg/dL, and when compared, survival was greater with *p* > 4 mg/dL (Figure 1D), with statistically significant differences (Log Rank = 5.274, *p* = 0.022).

The lower part of Figure 1 shows deaths and death percentages at 12, 24 and 36 months.

### 3.5. Cox Regression

Table 5 shows raw and adjusted all-variable Cox regression.

Univariant analysis showed significance for age, serum albumin, heart failure and P. Adjusted model analysis, kept significance for age, serum albumin, P and the percentage of proinflammatory monocytes CD14+/CD16+.

The increase in age and the percentage of CD14+/CD16+ proved to be mortality predictors, being that P and albumin increase protective variables. In this sense, for each one-year increase mortality showed a 5.6% growth whilst for each g/dL of serum albumin decrease, mortality increased 5.6 times. For each 1% increase in the number of CD14+/CD16+, there was a 2.2% increase in mortality and for every 1 mg/dL decrease in serum phosphus, mortality increased 2.5 times.

## 4. Discussion

We designed this study with the aim of comparing inflammation amongst hemodialysis patients with indwelling HDCs with nAVF-carrying patients. Patients with HDCs presented an increase in parameters measuring inflammation and microinflammation as compared with patients bearing a nAVF. Accordingly, CRP and serum ferritin but not albumin levels were significantly higher. Previous observational studies have already related CVCs as a significant influence in the inflammatory state of HD patients [15,16]. Wystrychowski et al. [17], have shown an improvement in inflammatory markers when HDC-dialyzing patients were changed to nAVF as dialysis access and the opposite.

Apart from classic humoral inflammation markers we have performed microinflammation studies, expressed by proinflammatory monocyte activation where we found a significant increase in the percentages of the CD14+/CD16+ subpopulation in HDC-bearing patients as compared to the ones with nAVF. Even though dialysis patients show elevated percentages of these cells respective from healthy subjects [18,19], CVCs seemingly increase cell inflammation compared with nAVFs. Coli et al. have already demonstrated an increase in both humoral and cellular inflammatory markers in HDC dialyzing patients respective of nAVFs confirming our results as catheters per se behave as independent inflammation promoters in HD patients [20], possibly related to biofilm formation in the inner catheter surface days after its insertion, as several studies have pointed out [21].

Although some authors have associated inflammation in patients with indwelling CVCs to catheter-related infections [22], only two patients in our group had suspected line infection with documented catheter-related bacteriemia supporting the role of catheters -in the majority of the non-infected catheters- in the subyacent inflammatory state as shown by Goldstein et al. [23].

Regarding the few complications recorded in patients with CVCt, taking into account that HD through a catheter has been associated with different complications, it should be noted that no deep vein stenosis was observed and the episodes of dysfunction were minor, being these results are consistent with those shown by Poinen K et al. (2019) [24] finding that CVC patients older than 79 have fewer catheter-related complications than younger populations [25]. Regarding the low number of infectious episodes in our cohort, we must state the complete adherence to the catheter-care guidelines in our patients [8], which we estimate crucial. The effect of the care provided in the prevention of infections—accounting for manipulation supported by the catheters along their use, especially when dysfunction appears—, tend to inadvertently pass in the majority of the studies [26], mostly in large population series, being difficult to find bibliography over the influence of nursing care in the prevention of catheter-related bacteriemias [27].

Looking into dialysis efficacy, patients dialized through a nAVF had better results than those with CVCs in all parameters despite different session length. In general, accordingly with previous studies [28,29], patients dialyzed through nAVFs receive better extrarenal depuration than those with HDCs. Nevertheless, in our sample 92% of HDC patients had a Kt/V > 1.3 and a Kt in men and women above 45 and 50 L, respectively as per recommended objectives of dialysis adequacy [30]. In addition, both groups of patients had a mean session duration of 250 min, a time higher than the recommendations of the European and Spanish guidelines (minimum of 12 h a week), which may explain the suitable analytical and efficacy parameters of the dialysis of the majority of the population studied, regardless of the VA used. The longer duration of the HD session has been related to better clinical results and higher survival even independently of Kt/V [31], with a session lasting less than 4 h being associated with a 42% increase in mortality [32]. Clearly then, there was no “underdialysis” which could explain this variable´s absent influence in mortality in opposition with other authors’ descriptions [33]. The ICC had similar scores within both groups, supporting the study´s results indicating appropriate matching for the proposed purposes. Taking into account that age, sex and comorbidity play an essential role in Health-Related Quality of Life (HRQoL) in HD patients [34], it would have been interesting to compare this variable between both groups, taking advantage of the matching of the same, since in previous studies worse HRQoL has been reported in patients who underwent dialysis using a CVC compared to those who did so using an AVF, although patients with a catheter had greater comorbidity [35]. We neither found differences in total hospital days during follow up, although HDC-carrying patients had greater hospital days unlike recent communications stating more episodes in shorter periods [36].

Another outcome was based on the comparison between both group´s survival and the analysis of factors impacting mortality amongst groups. When mortality was directly compared, patients with CVC´s presented proportionally more deaths at 12, 24 and 36 months, with an 11% excess mortality at the end of the follow up despite non statistically significant differences found due to the small sample size. There were neither differences in mortality between men and women nor between diabetic and non-diabetics. On the contrary, patients with serum-phosphate <4.0 mg/dL had greater mortality, with significant differences when compared with patients having >4.0 mg/dL. In the raw Cox proportional risks analysis, age, serum albumin, ICC and serum phosphate showed statistical significance. After adjusting for all variables, increasing age, CD14+/CD16 monocytes percentage, decreased serum albumin and phosphate remained in the model as predictors of mortality.

Increasing age and inflammatory condition as well as hypoalbuminemia and hypophosphatemia have been associated with mortality in dialysis patients by different authors and have also been postulated as independent interrelated predictors [37]. In this sense, in our study, VA did not work as a mortality predictor after adjusting for all variables. The explanation for these findings may be put in relation with the basal homogeneity between both groups of patients and in their age (75% > 70 years-old). When adjusting as possible baseline conditions amongst nAVFs with HDCs, VA does not seem to be the direct cause of mortality but an inflammation inducer indirectly responsible for malnutrition and greater mortality [38].

During the decade 2000–2010, several studies proved a greater mortality for patients with indwelling CVCs as compared to nAVF bearing patients [10,11,12,39,40] although there was a European investigation by Di Iorio et al. [41] claiming absent differences in mortality after adjusting by age, sex, poor nutritional status, diabetes, hemoglobin, albumin and other comorbidities. Later, Ravani et al. [42] in 2013, developed a meta-analysis including 62 studies totaling 586,337 patients showing an excess of all-cause mortality in HDC patients versus the group dialyzing through nAVFs recognizing a high risk of selection bias as a major weakness of their work. More recently, De Clerk et al. [43] in another European study found a 39% reduction in mortality ratio in nAVF-related hemodialysis compared to CVCs associated hemodialysis that—nevertheless—lost statistical significance after correcting for variables such as serum albumin, CRP or hemoglobin which suggests non-vascular access related mortality behind this study. Moreover, the cohort of patients included in this work presented a low ratio of catheter-related bacteriemias supporting the cardinal role of a continued adherence to CVC management best practice guidelines and of specialized dialysis nursing cares in this setting.

It may be hypothesized that as currently published studies approaching the involvement of CVCs in the mortality of hemodialysis patients are based in observational studies, this makes them prone to fall into a selection bias as there is a lack of better evidence coming from published randomized controlled trials absent so far in the literature.

It is possible that many patients end up with a HDC vascular access instead of a nAVF due to their more compromised condition. In this sense, Quinn et al. [44] found that the excess in mortality associated with CVCs could be not related to direct VA complications but to residual confusion, non-measured comorbidities or treatment-selection bias.

Moreover, Brown et al. [45] observed that incident hemodialysis patients starting through a HDC due to nAVF failure had a decreased mortality as compared to straight forward HDC starters arguing that these could explain up to two thirds of the benefit in mortality recorded in nAVF-based dialysis. This suggests that there are several factors linked to patients with a specific clinical profile that end up with an indwelling HDC due to the difficulties in nAVF creation [46].

Besides this, the increase in nAVF use—even in patients at risk of nAVF insufficient maturation—has yielded a greater chance of nAVF primary failure adding up complications and hospital days for this reason and therefore, catheter use continues to be a necessity in elder or comorbid patients unsuitable for nAVF creation or maturation making compulsory individualized nAVF referrals [47].

As main limitations of this study we must point out the fact of being a single center work which, added to the matching by mortality-influential variables, has enabled a small sample size. Nevertheless, this limitation also constitutes a main strength providing a very homogeneous cohort and the opportunity to compare two groups of patients with very similar baseline features—differing only in the VA—which undoubtedly strengthens its internal validity.

## 5. Conclusions

As a summary, we can conclude that the patients dialyzing through a tunneled CVC present a high degree of inflammation compared to the patients dialyzing through a nAVF. However, VA does not seem to directly influence mortality when patients are matched by the same covariables, at least in this group of patients. In this sense, in our department we continue referring all patients for nAVF creation although in elder patients with a short life-expectancy or severe comorbidities complicating nAVF creation or maturation, the use of HDCs must not be discarded. In these cases, it must be done with an emphasis on the prevention of eventual infectious complications and under a strict follow up of best catheter care and handling recommendations from specialized guidelines.

## Figures and Tables

**Table 1:** Baseline values for the paired variables and the base aetiologies of the total number of patients and of both groups of patients.

	Total Patients (*n* = 108)	AVF (*n* = 54)	VCC (*n* = 54)	*p*-Value
Age (years)	79 (70–83)	79 (70–83)	79.5 (69–84)	0.848
Sex (women)	60 (56%)	30 (56%)	30 (56%)	
Time on HD regimen (months)	62.6 ± 39.5	62.3 ± 39.6	62.8 ± 39.8	0.952
Presence of diabetes	42 (39%)	21 (39%)	21 (39%)	
Dialysis Technique Mode				
HD mode	80 (74%)	29 (31%)	51 (69%)	*
OL-HDF	28 (26%)	25 (89%)	3 (11%)	*
CKD aetiology				
CKD of unknown origin	45 (42%)	25 (46%)	20 (37%)	*
Diabetic nephropathy	22 (20%)	11 (20%)	11 (20%)	*
Glomerulonephritis	11 (10%)	5 (9%)	6 (11%)	*
Polycystic	11 (10%)	5 (9%)	6 (11%)	*
Interstitial	7 (7%)	3 (6%)	4 (8%)	*
Vascular	4 (4%)	2 (4%)	2 (4%)	*
Other	8 (8%)	3 (6%)	5 (9%)	*

Abbreviations: AVF: autologous arteriovenous fistula; VCC: venous central catheter; HD: hemodialysis; OL-HDF: on-line hemodiafiltration; CKD: chronic kidney disease. Note: Age: median and Q1–Q3; Time in HD: mean ± standard deviation; CKD aetiology: in the columns of patients with AVF and CVCt, the percentages with respect to the total number of patients in each group. * No interest in statistical comparison.

**Table 2:** Comparison between patients with AVF and VCC of the variables of inflammation and microinflammation.

	Total Patients (*n* = 108)	AVF (*n* = 54)	VCC (*n* = 54)	*p*-Value
CRP (mg/L)	4.9 (2.4–11.4)	3.4 (1.5–7.9)	7.4 (3.6–17.6)	0.002
Serum albumin (g/dL)	3.7 (3.4–3.9)	3.8 (3.5–3.9)	3.7 (3.3–3.9)	0.078
Serum ferritin (ng/dL)	540.8 ± 353.2	455.2 ± 352.7	625.5 ± 335.9	0.011
% CD14+/CD16+ monocytes	47.4 ± 12.3	43.6 ± 12.7	51.2 ± 10.9	0.001
% CD14+/CD16- monocytes	26.8 ± 12.3	27.4 ± 13.1	26.1 ± 11.7	0.595

Abbreviations: CRP: C-reactive protein; CRP and serum albumin: median and Q1–Q3; Serum ferritin, %CD14+/CD16+ monocytes, %CD14+/CD16- monocytes: mean ± standard deviation (SD).

**Table 3:** Comparison between patients with AVF and VCC of the analytical values studied, dialysis efficacy variables, comorbidity and days of hospitalization.

	Total Patients (*n* = 108)	AVF (*n* = 54)	VCC (*n* = 54)	*p*-Value
SBP (mmHg)	140.1 ± 28	145.2 ± 27	135 ± 28	0.069
DBP (mmHg)	71.3 ± 17	71.7 ± 14	71 ± 19	0.806
Haemoglobin (g/dL)	11.1 ± 1.2	11.4 ± 1	10.9 ± 1.5	0.55
Phosphate (mg/dL)	4.0 ± 1.0	3.8 ± 1.0	4.2 ± 1.1	0.083
Potassium (mEq/L)	5.1 ± 0.8	4.8 ± 0.8	5.4 ± 0.8	0.000
PTH pg/mL	229.6 (141–365)	223.6 (138–346)	276.2 (142–412)	0.588
HD session duration (minutes)	250 (245–250)	250.3 (245–250)	249.3 (245–252)	0.564
Volume of treated blood (liters)	94.1 ± 19.4	102.3 ± 19.5	85.8 ± 15.4	0.000
Blood flow (mL/min)	388.7 ± 75.8	421.2 ± 75.2	354.9 ± 54.1	0.000
Kt (liters)	52.8 ± 8.9	57.5 ± 7.3	48.4 ± 7.8	0.000
Kt/V	1.9 ± 0.5	2.0 ± 0.5	1.74 ± 0.5	0.005
CCI (points)	7 (6–9)	7 (6–9)	7 (6–9)	0.899
Hospitalization (days)	8 (0–19)	7 (0–18.2)	9 (0–25)	0.526

Abbreviations: AVF: autologous arteriovenous fistula; VCC: venous central catheter; SBP: Systolic Blood Pressure; DBP: Diastolic Blood Pressure; PTH: parathyroid hormone; CCI: Charlson Comorbidity Index; HD: haemodialysis; Kt: urea clearance*dialysis time; Kt/V: urea clearance*dialysis time/urea volume of distribution. SBP, DBP, Haemoglobin, phosphorus, potassium, volume of blood treated/session, dialysis blood flow, Kt, Kt/V: mean ± standard deviation. PTH, Duration of the HD session, ICC, days of hospitalisation: median and Q1–Q3.

**Table 4:** Causes of death according to vascular access.

	Total Patients (*n* = 108)	AVF Deaths (*n* = 21)	VCC Deaths (*n* = 27)
Undetermined cause	10 (21%)	6 (29%)	4 (14%)
Septicemia	5 (11%)	1 (4%)	4 (14%)
Other causes of heart failure	5 (11%)	4 (19%)	1 (4%)
Stroke	5 (11%)	1 (4%)	4 (14%)
Mesenteric infarction	4 (8%)	1 (4%)	3 (11%)
Malignant disease	3 (6%)	1 (4%)	2 (7%)
Other bleeds	3 (6%)	2 (10%)	1 (4%)
Lung infection	3 (6%)	1 (4%)	2 (7%)
Treatment interruption	2 (4%)	1 (4%)	1 (4%)
CRP of unknown origin	2 (4%)	2 (10%)	
Epileptic status	1 (2%)		1 (4%)
Acute myocardial infarction	1 (2%)		1 (4%)
Cachexia	1 (2%)		1 (4%)
Subdural haematoma	1 (2%)		1 (4%)
Septic shock	1 (2%)		1 (4%)
Peritonitis	1 (2%)	1 (4%)	

Abbreviations: AVF: autologous arteriovenous fistula; VCC: venous central catheter. Note: in the columns for AVF and CVVCt, percentages are indicated with respect to the total number of deceased patients in each group.

**Table 5:** Cox regression: crude and adjusted estimates for the study variables.

	Crude Estimate			Adjusted Estimate		
	HR	95% CI	*p*-Value	HR	95% CI	*p*-Value
Age	1.064	1.024–1.106	0.001	1.056	1.015–1.098	0.007
Sex (ref. woman)	1.174	0.666–2.069	0.579			
Vascular access (ref. AVF)	1.458	0.824–2.579	0.195			
Diabetes (ref. absence of diabetes)	0.855	0.476–1.533	0.598			
SBP	0.998	0.988–1.008	0.673			
DBP	0.984	0.967–1.003	0.094			
Kt/V	1.314	0.735–2.349	0.357			
Kt	0.974	0.944–1.005	0.094			
HD session duration	0.989	0.970–1.008	0.249			
Volume of treated blood	0.995	0.980–1.010	0.520			
Blood flow	1.000	0.996–1.004	0.928			
CRP	1.005	0.995–1.016	0.306			
Serum albumin	0.466	0.244–0.890	0.021	0.435	0.224–0.845	0.014
Serum ferritin	1.001	1.000–1.001	0.113			
CCI	1.165	1.023–1.327	0.021			
HD weather	1.005	0.998–1.012	0.165			
% CD14+/CD16+	1.012	0.989–1.036	0.317	1.022	0.997–1.047	0.089
% CD14+/CD16-	0.994	0.972–1.018	0.635			
Haemoglobin	0.932	0.749–1.159	0.534			
Serum phosphorus	0.687	0.520–0.909	0.009	0.750	0.569–0.989	0.041
Serum potassium	0.832	0.586–1.180	0.302			
PTH	1.000	0.999–1.001	0.507			
Length of stay	0.996	0.976–1.016	0.684			
Dialysis mode	0.706	0.352–1.418	0.328			

Abbreviations: HR: hazard ratio; CI: confidence interval; AVF: autologous arteriovenous fistula; SBP: Systolic Blood Pressure; DBP: Diastolic Blood Pressure Kt: urea clearance*dialysis time; Kt/V: urea clearance*dialysis time/urea volume of distribution; HD: haemodialysis; CRP: C-reactive protein; CCI: Charlson Comorbidity Index.; PTH: parathyroid hormone.

## Data Availability

Data are available upon reasonable request.

## Supplementary Materials

The following are available online at https://www.mdpi.com/article/10.3390/ijerph18147605/s1, Figure S1: Representative flow cytometry analysis.
