# Supplementary material for: Influence of Tunneled Hemodialysis-Catheters on Inflammation and Mortality in Dialyzed Patients
Source: Int J Environ Res Public Health. 2021 Jul 16;18(14):7605. doi: 10.3390/ijerph18147605 (PMC8304695; doi:10.3390/ijerph18147605)
Supplement: Supplementary file 1 [file ijerph-18-07605-s001.zip › ijerph-1292171-supplementary.pdf]

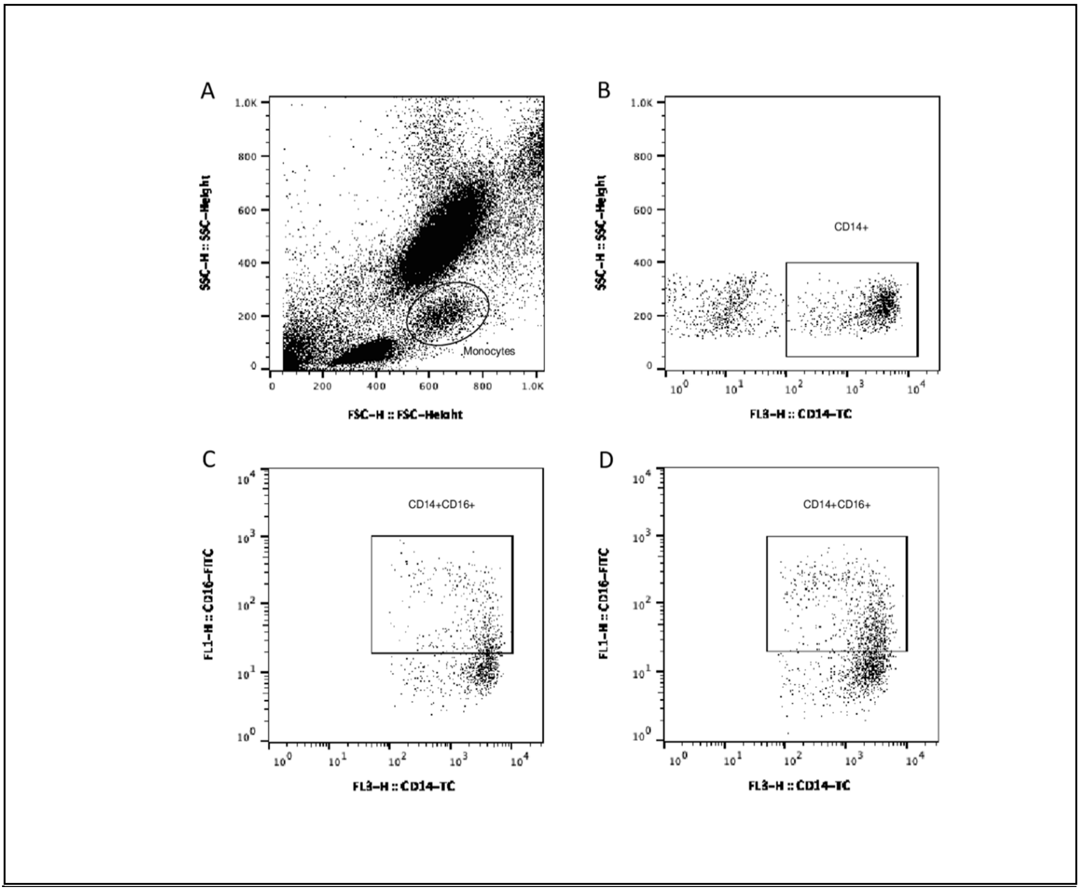

Figure S1. Representative flow cytometry analysis. (**A**) Monocytes were identified based on their forward scatter (FSC)-Height and side scatter (SSC)-Height characteristics. (**B**) Monocyte gate was further visualized in an SSC-Height versus CD14-TC plot to discriminate monocytes of other leukocytes that are not of interest. Subsequently, CD14+CD16+ monocytes within CD14+ population were assessed using CD16-FITC versus CD14-TC plot. Representative dot plot CD14+CD16+ expression in (**C**) AVF and (**D**) catheter CKD patients.
